# Diurnal variations of cloud and relative humidity profiles across the tropics

**DOI:** 10.1038/s41598-019-52437-6

**Published:** 2019-11-05

**Authors:** H. Chepfer, H. Brogniez, V. Noel

**Affiliations:** 1 LMD/IPSL, Sorbonne Université, Ecole Polytechnique, CNRS, Paris, France; 2 LATMOS/IPSL, UVSQ Université Paris-Saclay, Sorbonne Université, Guyancourt, France; 3 LA, CNRS, Toulouse, France

**Keywords:** Atmospheric science, Applied physics

## Abstract

Even though the diurnal cycle of solar forcing on the climate system is well defined, the diurnal evolutions of water vapor and clouds induced by the solar forcing are not yet established across the tropics. Here we combine recent satellite observations of clouds profiles and relative humidity profiles to document the diurnal variations of the water vapor and clouds vertical distributions over all the tropics in June-July-August. While the daily mean water vapor and cloud profiles are different between land and ocean, their diurnal variations with respect to their daily means exhibit similar features. Relative humidity profiles and optically thin cloud fraction profiles vary together which maximize during night-time in the entire troposphere and a minimize in day-time. The fraction of optically opaque clouds peak in the free troposphere in the early afternoon, transforms into a high altitude positive anomaly of optically thin clouds from nightfall to sunrise. In addition, land regions exhibit a daily low thin cloud positive anomaly, while oceanic regions exposed to subsidence air motions exhibit positive anomalies of opaque clouds in the lower atmosphere during the second half of the night, which grow until sunrise.

## Introduction

Every day, the tropics are exposed to large variations of incoming solar radiation, from zero at nighttime to about 1300 W/m^2^ at noon. Hence, in the tropical atmosphere, water in all its forms (vapor, liquid, solid) breathes at a diurnal rhythm: the amount and spatial distribution of water vapor, clouds, and precipitation can vary throughout the day. The surface and the atmosphere respond to the strong diurnal solar forcing by continuously adjusting the energy exchanges between the surface and the atmosphere, and between the different compartments of the atmosphere. The surface absorbs part of the incoming solar radiation during day-time and re-emits continuously this energy toward the atmosphere. The associated variations in the surface-atmosphere coupling and boundary layer stability influence the evaporation of surface water in the atmosphere and the formation of clouds.

Previous studies have established that in the lower and upper troposphere cloud and water vapor diurnal variations are driven by different physical mechanisms.

In the lower troposphere over ocean, the marine stratocumulus exhibits a well-understood strong diurnal cycle with a maximum coverage during the early morning before sunrise^1^. However, there have been limited studies on understanding the diurnal cycle of continental stratocumulus and marine cumuli^2–4^.

In the free troposphere, the cloud diurnal cycle differs dramatically from that in the low troposphere^5^. Here, the cloud diurnal cycle is driven by convective heating from below and exhibits a maximum at night^6^. Over land, the diurnal cycle of the convective cloud has been attributed to a direct thermodynamic response to the strong diurnal cycle of the land surface temperature^7^, while over ocean the physical mechanisms behind the diurnal cycle of convective clouds remain poorly understood (e.g.^8–10^).

These gaps in our current understanding of how water vapor and clouds diurnal cycles vary together are largely nourished by the lack of a comprehensive observed picture of these characteristics in the lower troposphere and free troposphere over the entire tropics.

Hence, while the diurnal variations of the total precipitable water and cloud amounts across the tropics have been established (e.g.^11–13^), most of the satellite-based studies^14–19^ and most model studies^4,10,20–24^ do not include the detailed vertical dimension necessary to robustly distinguish the diurnal cycles of humidity^25–28^ and clouds in the lower troposphere and the free troposphere. Furthermore, satellite-based climatologies are usually less robust over land than ocean, which limits the possibility to contrast the diurnal cycle of clouds and water vapor over the different surfaces. Field experiments and ground-based observations^2,3,5,29–32^ have provided substantial information on the diurnal evolution of the vertical profiles of water vapor and clouds but these are confined regionally and focus on the processes that drive the lower troposphere.

Because the mechanisms operating in the lower troposphere and in the free troposphere are distinct, a comprehensive understanding of the diurnal evolutions of moisture and clouds over the whole tropics requires observations that distinguish unambiguously the evolutions in the free troposphere from the ones in the lower troposphere. In addition, these observations of water vapor and clouds need to be reliable above both ocean and land in order to robustly examine their differences and similarities. Such analysis has not been performed because the relevant observations were missing. Recently, advanced space-borne instruments have collected useful measurements that can fill this knowledge gap and help build a more comprehensive picture.

Here we take advantage of three space-borne instruments that observe the relative humidity profiles^33^, the cloud profiles^34,35^, and the top of the atmosphere radiation^36,37^ across the tropics. The low orbit of the Megha-Tropiques satellite samples the diurnal variations of both RH profiles and top of the atmosphere radiation, while the cloud profiles are documented thanks to a lidar onboard the International Space Station. See the Data and Methods section for a description of instruments, data and analysis.

## Results

### Tropical water vapor and cloud profiles

**Figure 1:**
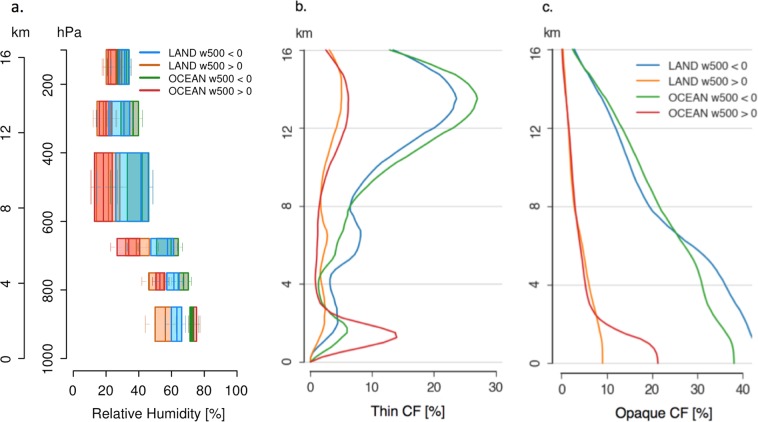
Daily mean profiles over 24 hours of (**a**) Relative humidity (**b**) Fraction of optically thin cloud with optical depth less than 3 to 5 (**c**) Fraction of clouds opaque to radiation with optical depth larger than 3 to 5, over four tropical areas namely land subsidence (omega500 > 0), land ascent (omega500 < 0), ocean subsidence (omega500 > 0) and ocean ascent (omega500 < 0). Built from low orbiting space observations of relative humidity by SAPHIR/MeghaTropique and opaque/thin cloud profiles by CATS/ISS during June-July-August 2015-2016-2017 over 30°S to 30°N. The vertical wind speed at 500hPa (omega500) is monthly mean from ERA5.

Figure 1 shows the daily mean (over 24 hours) profiles of RH, opaque clouds and thin clouds in different tropical areas (see Methods). These newly combined observations show distinct characteristics in the lower troposphere and in the free troposphere.

First in the lower troposphere (P > 800hPa), describing the boundary layer, the RH is lower (<60%) over land in subsiding regions while it is slightly larger over land in ascending regions (RH 65%), and closer to saturation over all oceans (RH >70%). The thin cloud fraction profiles follow the changes in the moisture profiles: the region with lowest RH produces the smallest thin cloud fractions (CFthin < 3%) and the region with largest RH is accompanied with the largest thin cloud fraction (CFthin > 13%). The RH and cloud profiles in the boundary layer depend primarily on the surface water availability (land vs. ocean), and secondly on the atmospheric circulation (omega500 > 0.vs. omega500 < 0).

The free troposphere (P < 600hPa) contrasts with the lower troposphere. In the free troposphere, the subsiding regions show a rather weak RH (~20%). These areas are known to be associated to longwave cooling rates that increase as the free troposphere becomes drier^38,39^. The dry free troposphere is almost cloud free (CFthin < 10% and CFopaque < 10%), with a low z-opaque (~3.1 km, Table 1) and a warm surface temperature (299.9 K) leading to a large OLR (269 W/m^2^). The altitude of opacity (z-opaque) is the height at which emissivity integrated from the top of the atmosphere equals almost one^40^. Unlike the subsidence regions, the ascending regions have a moister free troposphere containing much more thin clouds (CFthin > 20%) and more opaque clouds at high altitude (CFopaque > 10%). There z-opaque reaches ~8 km instead of 3 km (Table 1): the moister atmosphere raises the altitude of opacity of the atmospheric column and thus reduces the OLR (Table 1). In the free troposphere (P < 600hPa), the profiles of moisture and clouds are primarily constrained by the atmospheric circulation (ascending.vs. descending), while the surface water availability (land.vs. ocean) plays a secondary role.

**Table 1:** Daily mean values over four tropical areas, namely land subsidence (omega500 > 0), land ascent (omega500 < 0), ocean subsidence (omega500 > 0) and ocean ascent (omega500 < 0). “z-opaque” corresponds to the altitude where the atmosphere emissivity from the top-of-the atmosphere is about 1. The OLR and Absorbed SW are built from ScaRaB/Megha-Tropique, the Skin Temperature from ERA5, the others variables from CATS/ISS. Based on June-July-August 2015-2016-2017 over 30°S to 30°N.

	Land subsidence	Land ascent	Ocean subsidence	Ocean ascent
OLR (W/m^2^)	275	233	269	238
Absorbed SW (W/m^2^)	302	276	330	309
Skin Temperature (K)	298.8	298.8	299.9	298.0
Cover Opaque clouds (%)	8.3	30	17.5	27.4
Cover Thin clouds (%)	22.8	40.3	32.2	42.1
Cover Clear sky (%)	68.9	29.7	50.3	30.5
z-opaque (km)	5.7	7.9	3.1	8.2

Because of these clear separations between the daily mean profiles in the four areas, we further examine the diurnal variations for each of them. The oceanic subsidence region includes at least two different regimes: the marine stratocumulus regime such as offshore California or Peru, and the near clear-sky regime such as under subtropical highs. These two regimes should undergo different diurnal variations because there is a difference if the sunlight can reach or not the ocean surface. Therefore the oceanic subsidence region is splitted into two different categories: a strong subsidence category (mid-tropospheric vertical velocity omega500 > 30 hPa/day) containing mostly stratocumulus decks and a moderate subsidence category (0 < omega500 < 30 hPa/day) containing mostly trade cumulus clouds with intervals of cloud-free areas.

### Diurnal cycles of moisture and clouds profiles

**Figure 2:**
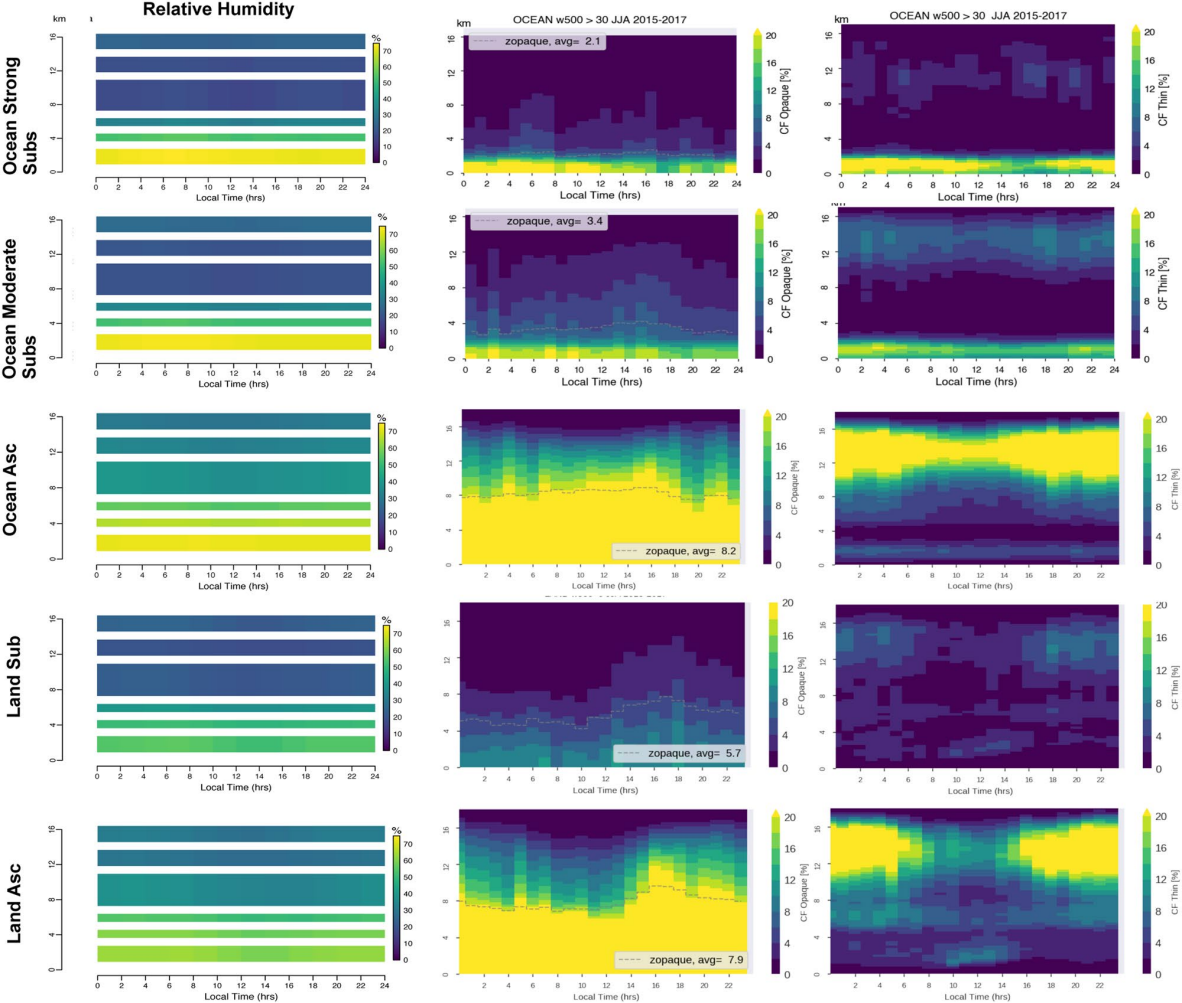
Diurnal evolutions of the profiles of relative humidity (first column), opaque clouds (second column) and thin clouds (third column), over oceanic strong subsidence (omega500 > 30 hPa/day, first row), oceanic moderate subsidence (0 < omega500 < 30 hPa/day, second row), oceanic ascent (third row), land subsidence (fourth row), land ascent (fifth row). “z-opaque” level corresponds to the altitude where the atmosphere emissivity from the top-of-the atmosphere is about 1. The cloud fractions (opaque or thin) are masked in grey when they are computed from less than 1500 profiles.

Figure 2 shows how the atmospheric water, clouds and relative humidity, evolves vertically during the day. The range of changes along the day is generally small: the absolute variation of RH is typically at most 20% at any given level of altitude along the day. Similarly, the absolute variations of opaque cloud fraction and thin cloud fractions reach at most 10% at any given altitude. In others words, Fig. 2 shows that the strong diurnal forcing exerted by the incoming solar radiation (from 0 at night to more than 1300 W/m^2^ at noon) has a small impact on the RH and clouds profiles compared to the other controlling factors such as the surface water availability and atmospheric circulation.

**Figure 3:**
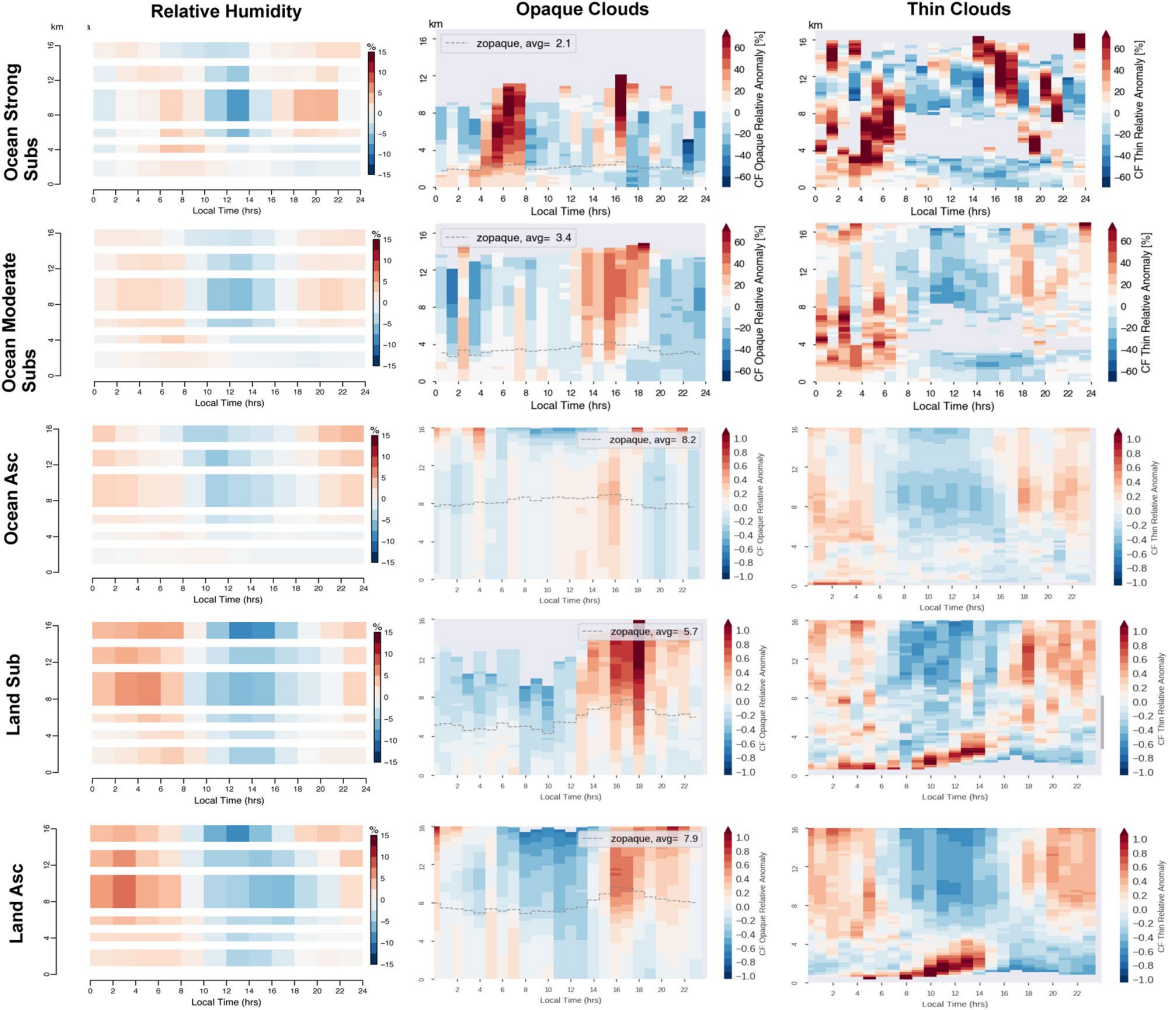
Same as Fig. 2 but in relative anomalies with respect to the daily mean at each level of altitude. e.g. [CFthin(z,t) − <CFthin(z)> ]/ <CFthin(z)> where CFthin(z,t) is the hourly mean shown in Fig. 2 and < CFthin(z) > is the daily mean shown in Fig. 1. The relative anomalies are masked in grey when the number of profiles used to compute CFthin(z, t) is lower than 1500 and CFthin(z, t) <1%.

How the RH and clouds respond to the solar radiation diurnal forcing is revealed by the use of relative anomalies with respect to the daily mean (Fig. 3): for a given altitude the relative anomaly represents the difference between the hourly value (Fig. 2) and the daily average, divided by the daily average. This informs on the amplitude of variation of the parameters with respect to their base state.

The five regions present roughly the same behavior. A small positive RH relative anomaly (less than +15% of its daily mean) develops during the second-half of the night (0–6 am) in the boundary layer and in the free troposphere and is associated to a positive anomaly of thin clouds in the entire troposphere. During day-time, a negative RH relative anomaly occurs with a maximum around 12 am in the free troposphere; it is associated to a negative thin cloud fraction relative anomaly in the free troposphere.

**Figure 4:**
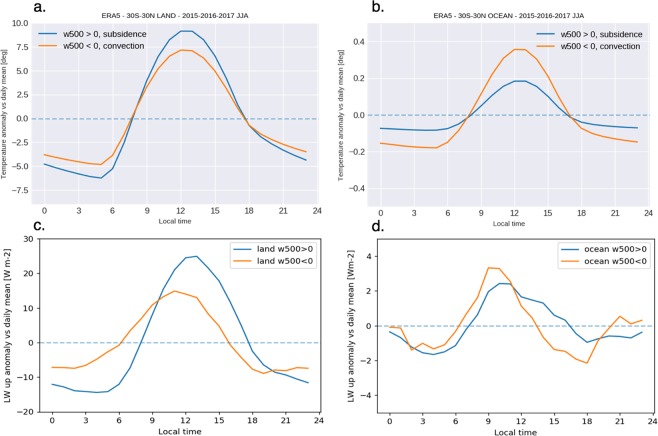
Diurnal evolutions of the skin Surface temperature over (**a**) land and (**b**) ocean, and Outgoing long-wave radiation over (**c**) land and (**d**) ocean. Plots show the absolute anomalies with respect to the daily means built from hourly ERA-5 skin surface temperature and from ScaRaB/MeghaTropique OLR during June-July-August 2015-2016-2017 over 30°S to 30°N. The vertical wind speed at 500hPa (omega500) is monthly mean from ERA-5.

The joint evolution of RH with thin clouds is likely driven by the diurnal evolution of the surface temperature (Fig. 4) rather than by a change in the amount of water vapor in the atmosphere. Indeed, when the temperature decreases and reaches a minimum in the late night, RH increases by definition, and additional thin clouds are formed.

Interestingly the opaque clouds relative anomaly does not seem always tied to RH. The opaque clouds positive anomaly is maximum at 4 pm in the free troposphere and disappears during the early night. The deep convection develops over both land and ocean in the early afternoon (1–4 pm) even over ocean located far from continents (not shown). The OLR (Fig. 4) reaches a maximum when the free troposphere clouds anomalies are at their minima: at 9am over the ocean and at 12 am over land. Conversely the OLR reaches a minimum when the cloud anomalies are at their maxima. Although all the tropics share this common behavior, different regions show some differences in their diurnal cycles in the boundary layer.

The land regions exhibit a specific low altitude diurnal cycle that is not present over the ocean. A pronounced positive relative anomaly in low level thin clouds (+100% of its daily mean) shows up after sunrise and rises up from the surface while remaining within the boundary layer. RH also exhibits a positive relative anomaly before 10 am, which is probably causing the thin cloud anomaly.

The strong subsidence oceanic region exhibits a specific positive relative anomaly of opaque clouds (about 20%) during the second half of the night(0–8 am) within the boundary layer, which does not occur in the others regions. This positive anomaly of opaque clouds is initially located in the boundary layer, and extends to the free troposphere later between 4 and 8 am. A weak positive relative anomaly of RH (about 5%) develops close to the surface between 4 and 8 am. This shows how water accumulates in all its forms (vapor, thin clouds, opaque clouds) during nighttime in the boundary layer until the sun rises, under the influence of a warm ocean and a strong subsiding free troposphere motion above. This mechanism agrees with the current understanding of the stratocumulus diurnal cycle (e.g.^1^).

Also, ocean regions characterized by ascent show a positive anomaly of thin clouds within the first km above the surface during the late night.

These results establish a comprehensive observational picture of the diurnal cycles of clouds and RH profiles across the tropics and the specificities for ascent and subsidence over land and ocean. Because clouds and RH distributions also influence the surface temperature, we next examine the diurnal variation of the surface temperature in each region and their relationship with the diurnal variation in clouds and RH.

### Linking surface temperature and atmospheric diurnal variations

The daily evolution of the surface temperature differs in amplitude and shape from one region to another (Fig. 4). These evolutions are primarily driven by the heat capacity of the surface, leading to larger amplitude of the temperature diurnal variations over land than ocean. The atmosphere also plays a role on the temperature, through complex boundary layer processes and cloud shading. Hereafter we examine only the radiative effect of the diurnal cycle of RH and clouds on the surface temperature.

The amplitude of the temperature diurnal cycle – defined as the difference between the max and min temperatures along the day- is influenced by the amount of water vapor in the boundary layer and by clouds. Clouds reflect solar incoming radiation and both water vapor and clouds efficiently warm the surface radiatively in the infrared and weaken the amplitude of the temperature diurnal variation produced by the solar incoming variation. Over land, the boundary layer exhibits a moister RH profile over regions characterized by ascent (Fig. 1) contributing to a smaller temperature amplitude diurnal variation (12 K) compared to descent (15 K, Fig. 4). Overall, the subsidence ocean region has the smallest temperature diurnal variation (0.3 K) first because the ocean heat capacity is large compared to the land one, and to a lower extent because it presents a persistent increasing positive anomaly of opaque clouds during the second half of the night that maintains a surface warming during the second half of the night until sunrise.

The asymmetry between the morning evolution and afternoon evolution of the temperature seems mostly depending on the free troposphere RH and clouds (Fig. 4). The surface temperature is minimum at the end of the night (5am) because the surface loses radiation by emission all night long. The maximum of absorbed solar radiation occurs at noon and consequently the surface temperature is maximum right after noon. In the free troposphere, the opaque clouds formed in the afternoon and the persistent thin clouds that last later during the night, help maintain a high surface temperature in the afternoon, compared to the morning when free troposphere cloud fraction is very low. The numerous afternoon free tropospheric clouds slow the cooling of the surface between noon and midnight and likely cause the asymmetry in the surface temperature evolution. The shape of the temperature diurnal cycle is influenced by the afternoon opaque clouds anomaly in the free troposphere (Fig. 4). Moreover, the lowest RH and most cloud-free tropical region (subsidence land) features a slow afternoon cooling due to the largest relative increase (+100% of its daily mean) of opaque clouds in the free troposphere in the afternoon (3–8 pm) which may partially result from a positive anomaly of thin clouds in the boundary layer earlier (8 am–3 pm)

This study establishes an observationally based link across all the tropics 1) between the amplitude of the diurnal cycle of surface temperature and the RH-cloud persisting within the boundary layer (P > 800 hPa) during the second half of the night, and 2) between the speed of the temperature decrease and free troposphere (P < 800 hPa) opaque clouds during the afternoon.

## Discussion

Even if the observations used here provide new information, they have some limitations. Due to the design of the microwave sounder, the RH profiles cannot be retrieved when the atmospheric column contains precipitation and large ice crystals^41–43^. Thus the range of the RH relative anomaly values presented in this work are likely biased toward low values as the column containing precipitation are expected to reach saturation. Another limitation is the inability of the space-borne lidar to detect thin clouds located below opaque clouds. It implies that the boundary layer thin clouds can be underestimated in regions where high-altitude opaque clouds are present. This masking effect is maximum in the afternoon over land regions characterized by ascent. By counting the number of fully attenuated lidar profiles, we found the boundary layer thin clouds fraction could be underestimated by at most 30% over those regions. Another limitation is the categorization in monthly mean of vertical pressure velocity. Even if a location is under monthly mean subsidence, storm systems that may be coupled with equatorial waves can overpass that location quasi-periodically within a month. For example, the higher amount of opaque clouds over oceanic regions characterized by strong subsidence (Fig. 3) is likely not a characteristic feature of the diurnal cycle in those locations but an artifact introduced by those transient storms.

Despite these limits, these newly combined instruments provide an observationally based comprehensive understanding of how the RH profiles, the clouds profiles, the radiation at the TOA and the surface temperature vary together along the day under the influence of changing incoming solar radiation, across the entire tropics. The advances include the first observation of the relative humidity diurnal cycle profile from the TOA down to the surface across the tropics including the relative humidity in cloudy portions of atmosphere. The microwave observations of RH used here are not limited to cloud-free regions like most previous studies (e.g.^36^). Another advance is the ability to describe the detailed vertical cloud profile – and distinguishing opaque and thin clouds – while most of the existing global-scale characterizations of the cloud diurnal cycle are limited to column-integrated variables. A final advance is the ability to characterize the clouds over land, thanks to the space lidar capability, while most of the previous satellite-based characterization of the cloud diurnal cycle are not reliable over land.

Overall, these observations present a new view of the cloud and RH diurnal cycle in the tropics (Fig. 3). In the low troposphere over oceanic subsidence, our results are consistent with the diurnal cycle expected for marine stratocumulus clouds (e g.^1^). They fill a gap in the observations of the diurnal cycle of low altitude clouds and humidity over land overall the tropics. In the free troposphere, they fill an observational gap between 4 km and 12 km while previous studies mostly focused on the upper troposphere only (e g.^10,44^). The results presented here indicate high opaque clouds peak close in time over ocean (3 pm) and land (5–6 pm). They also suggest that the diurnal cycle of the free tropospheric RH are similar over ocean and land. The free tropospheric RH tends to peak at night like the thin clouds, and it is minimum between 10am and 4 pm. Thin clouds remain all night long over the entire free troposphere.

Beyond this study, these observations contain likely valuable information for progressing on the current knowledge of the stratocumulus and shallow cumulus diurnal cycles. These observations could be used to test the physic of models, by evaluating jointly the simulated diurnal variations of RH and cloud profiles. At a global scale, while a recent study has evaluated the description of the cloud cover diurnal variation in climate models^23^, the joint variations of cloud profiles and RH profiles were not evaluated. At a regional scale, this provides also useful benchmarks to evaluate such description of the diurnal cycles in regional models.

## Data and Methods

In this study, we use observations from satellite instruments that (1) can document the vertical distribution of key components of the atmospheric water cycle, (2) can observe the same location at different local times of day. These instruments on-board Megha-Tropiques and the International Space Station (ISS), have low orbits that give them a good sampling of tropical latitudes. We focus on June-July-August 2015, 2016, 2017 and the 30°S-30°N region where both Megha-Tropiques and the ISS collected data.

### Water vapor profiles

Megha-Tropiques has been launched in 2011 on a 20°-inclination orbit and it is still operating today. It typically observes the same location between 3 and 5 times each day in the Tropical belt (30°N-30°S) and performs 14 revolutions per day. The orbit is also characterized by a 51-day precession cycle: then each day the 3 to 5 orbits come in spaced out packets that are 102 min to roughly 20hrs apart, with a regular backward shift in the local time of observation. This implies that a same location is observed at different local times during successive days^37,45^. By aggregating data collected during several days we can thus reconstruct the diurnal variation.

In this study we use the relative humidity (RH, in % with respect to liquid water) profiles derived from observations of the 183 GHz microwave sounder SAPHIR. While brightness temperatures are directly related to RH, deconvolving from temperature effects to get absolute humidity requires to include the temperature profiles from an external source. So, even if the specific humidity would present more interest for describing the water vapor diurnal cycle than RH, we focus here on RH which is the reliable quantity measured by SAPHIR.

The RH profiles are estimated using a multivariate retrieval scheme over 6 unevenly spaced atmospheric layers, defined from the observing channel’s sensitivity functions and ranging from 1000hPa to 100hPa (41 and references therein). Each profile corresponds to an atmospheric column of about 10-km diameter at nadir. Comparing the SAPHIR RH profiles against radiosoundings^41^ and an airborne Raman lidar^46^ has highlighted the overall good performances of the retrieval scheme, with root-mean-square errors below 12%. The limits of retrieval arise when dealing with multilayer situations, where the passive instrument fails to detect strong (80% to 10%) vertical humidity gradients.

### Opaque and thin cloud profiles

The cloud profiles used in this study are derived from the Cloud Aerosol Transport System (CATS) - ISS lidar^47^. CATS has collected almost 3 years of data between January 2015 and November 2017. It operated from the ISS, which orbits the Earth 16 times a day an altitude between 350 and 410 km and a 51.6° inclination. This low-Earth orbit means CATS could not track how the atmosphere changes diurnally over a fixed location on a particular day. However, as the ISS is non sun-synchronous like Megha-Tropiques, we can reconstruct a statistically representative view of the diurnal changes of the atmosphere over a given location, by aggregating CATS data collected over a long period at various local times during different days.

In this study, we use cloud detections from CATS Level 2 Operational products^34,35^ and apply the same cloud selection as in^6^. In addition, we classify clouds in two categories: opaque and thin (as in 48). This separation allows to directly relate clouds to their radiative effects. Opaque clouds, with large optical depths (>3), drive the longwave and shortwave cloud radiative effects at the top of the atmosphere^48,49^. In contrast, thin clouds with optical depths below 3 to 5 have less influence on radiation^48,49^. In the present study, we use the “opaque cloud” flag from CATS Level 2 data, and we defined z-opaque as the base of the lowest opaque cloud seen by the lidar^40^. z-opaque documents the lowest altitude that contributes to the outgoing long wave radiation^49^.

### Outgoing longwave radiation and surface temperature

We use the top of the atmosphere outgoing longwave radiation observed by the broad-band radiometer ScaRaB^32^ onboard Megha-Tropiques^33^.

In addition to the satellite data described here above, we also use the skin surface temperature from ERA5 at an hourly time resolution, and the vertical air wind speed at 500 hPa (omega500) from ERA5 at a monthly mean time resolution.

### Building the diurnal cycles

At first order at global scale, the spatial distributions of moisture and clouds are constrained by the amount of water available at the surface and by the large-scale atmospheric circulation. The surface water availability is limited over land while it is unlimited over ocean. The large-scale atmospheric circulation is dominated by the ascending and descending branches of the Hadley cell in the tropics. Therefore, we study the RH and cloud profiles separately over land and over ocean, and we distinguish areas under the influence of subsidence air, noted omega500 > 0, from areas under the influence of ascent, noted omega500 < 0. The *oceanic subsidence region* is where air goes down on a monthly average (omega500 > 0). It covers the largest area, almost 2/3 of the tropical ocean, and corresponds to the descending branch of the Hadley cell that limits the entrance of moisture at mid and high altitudes. This region contains mostly stratocumulus clouds and shallow clouds^1^. *The oceanic ascending region* (omega500 < 0) corresponds mostly to the Inter Tropical Convergence Zone: it contains the deep convective clouds and covers a smaller surface of the tropical belt. The *continental subsidence region* (omega500 < 0) and the *continental ascending region* (omega500 > 0) are smaller than the two oceanic regions.

Each month, we flag each 2° × 2° grid box as belonging to one of the region depending on the surface type (ocean or continent using the surface type included in the CATS product) and on the value of omega500. The gridboxes that include both land and sea are not used in the analysis.

For each grid-box of a given region, we keep all the individual observed parameter together with their associated local time (LT). Then we build a composite diurnal cycle for a given region by averaging according to the LT and for the months of interest (JJA 2015, 2016, 2017). This is done for all the data: RH profiles, outgoing long-wave radiation, opaque cloud profiles, thin cloud profiles, z-opaque, and surface temperature.

Moreover, we estimated the uncertainty on the cloud fraction diurnal cycle and on the relative diurnal anomaly of the cloud fraction following a bootstrapping methodology. It results that the uncertainties on the cloud fraction diurnal cycle are well distributed vertically and reach at most 0.3% for cloud fractions above 1%. Uncertainties on the relative diurnal anomalies of the cloud fraction are also well distributed vertically and reach at most 10% for the relative cloud fraction anomalies between −60% and +60%.
